# The relationship between corneal biomechanics and endothelial morphology: evidence from a Saudi cohort

**DOI:** 10.3389/fmed.2026.1715007

**Published:** 2026-04-30

**Authors:** Ali M. Alsaqr, Noura Al-ahmad, Meshary Alrumizan, Ali Abusharha

**Affiliations:** Optometry Department, College of Applied Medical Sciences, King Saud University, Riyadh, Saudi Arabia

**Keywords:** corneal biomechanics, corneal hysteresis, corneal resistance factor, endothelial cell density, intraocular pressure, Saudi population, specular microscopy

## Abstract

**Background:**

Corneal biomechanics and endothelial morphology are key to ocular stability, but their relationship in healthy adults remains understudied.

**Methods:**

In this cross-sectional study, 122 healthy Saudi adults aged 18–40 years were examined using the Ocular Response Analyzer (ORA) to measure corneal hysteresis (CH), corneal resistance factor (CRF), Goldmann-correlated intraocular pressure (IOPg), and corneal-compensated intraocular pressure (IOPcc). Corneal endothelial morphology and central corneal thickness (CCT) were assessed using non-contact specular microscopy, providing endothelial cell density, cell count, hexagonality, and morphometric indices. Measurements were obtained under standardized conditions. Associations between biomechanical, endothelial, and refractive variables were evaluated using ANOVA, Pearson correlation analysis, and multivariate regression models.

**Results:**

Males showed slightly lower CH, CRF, and endothelial indices, though differences were not significant. IOPg was significantly higher in hyperopes versus myopes (*p* = 0.014), while no refractive group differences were observed for CH, CRF, or IOPcc. CCT correlated moderately with CH (*r* = 0.40, *p* < 0.0001), CRF (*r* = 0.53, *p* < 0.0001), and IOPg (*r* = 0.50, *p* < 0.0001), while endothelial cell number (NUM) was negatively associated with IOPg (*r* = −0.23, *p* = 0.011) and IOPcc (*r* = −0.21, *p* = 0.021). Regression confirmed CCT as the strongest predictor of ORA outcomes, whereas endothelial and refractive parameters contributed minimally. Compared with international datasets, CH and CRF values in this Saudi cohort were broadly comparable, though inter-ethnic variations were noted.

**Conclusion:**

Corneal biomechanics in healthy adults are predominantly driven by central corneal thickness, with endothelial morphology exerting a secondary modulatory influence on pressure-related and viscoelastic responses. These findings provide foundational normative data with relevance for refractive surgery, glaucoma assessment, and regional screening practices.

## 1 Introduction

The cornea is a transparent, avascular tissue that contributes approximately two-thirds of the total refractive power of the eye and serves as its primary structural barrier. Optical clarity is maintained by the highly ordered collagen fibrils of the stroma and the pump–barrier function of the corneal endothelium, a non-regenerating monolayer of hexagonal cells that regulates stromal hydration via ionic transport. Compromise of endothelial structure or function leads to stromal edema, loss of transparency, and irreversible visual impairment (1).

Corneal biomechanics describe the elastic and viscoelastic properties of the cornea in response to internal (intraocular pressure, IOP) and external (applanation, air-puff) forces. These properties are commonly characterized by corneal hysteresis (CH), a measure of viscoelastic damping capacity and the corneal resistance factor (CRF), a surrogate of overall tissue rigidity measured by the Ocular Response Analyzer (ORA) and are influenced by demographics and geometry (2). Corneal biomechanical indices are shaped by multiple structural and physiological factors, particularly central corneal thickness (CCT), intraocular pressure (IOP), age, curvature, and hydration (3–5). Both CCT and IOP are major determinants of ORA-derived parameters, with several studies demonstrating a strong influence of IOP on measured biomechanical responses (6–8). In a previous study, CCT emerged as the strongest predictor within their cohort (9). CH and CRF also decline with age and generally increase with CCT (10, 11). Refractive error may also influence corneal biomechanics. High myopia has been associated with lower CH and CRF and with greater corneal deformability in some studies (12–14), although these relationships are less consistent in younger populations (15). Importantly, ethnic variation plays a substantial role in corneal biomechanics. Multi-ethnic studies demonstrate that Indian adults exhibit higher CH and CRF than Caucasians, who in turn have higher values than Chinese participants (16). Such findings highlight the necessity of population-specific normative data.

Although biomechanical indices primarily reflect stromal architecture, there is increasing interest in their relationship with the corneal endothelium. Some evidence suggests correlations between CH, CRF, and endothelial parameters such as endothelial cell density (ECD), hexagonality (HEX), and coefficient of variation (CV) (5). In disease states such as keratoconus and Fuchs endothelial corneal dystrophy, both biomechanics and endothelial morphology can deteriorate concurrently (17). Furthermore, genetic studies have identified loci, such as ANAPC1, that influence both ECD and CH, suggesting potential shared molecular mechanisms (1).

In Saudi Arabia, available research has reported baseline CH and CRF values in healthy individuals (10), but no studies have comprehensively investigated the relationship between corneal biomechanics and endothelial morphology in a healthy Saudi adult cohort. Given the ethnic variation in biomechanical properties and the clinical significance of both corneal systems, establishing a Saudi-specific profile is critical.

This study aims to investigate the relationship between corneal biomechanical properties and corneal endothelial morphology in a healthy Saudi adult population. Such data could contribute to improved screening accuracy for refractive surgery candidacy, glaucoma risk stratification, and early detection of corneal diseases. The primary objective was to determine whether endothelial morphology independently contributes to ORA-derived corneal biomechanical parameters in healthy Saudi adults. Secondary exploratory comparisons with previously published populations were included to contextualize the observed biomechanical profile.

## 2 Materials and methods

### 2.1 Ethical considerations

This cross-sectional, observational study was conducted at King Saud University, Riyadh, Saudi Arabia, between January 2025 and June 2025. All procedures were performed in accordance with the Declaration of Helsinki, and written informed consent was obtained from all participants. The study protocol received approval from the Institutional Review Board of King Saud University (Approval No. E-25-9590).

### 2.2 Sample size determination

Sample size estimation was performed *a priori* using G*Power software (version 3.1). Assuming a moderate correlation (*r* = 0.30) between corneal biomechanical and endothelial parameters, an alpha level of 0.05, and statistical power of 0.90, the required minimum sample size was calculated to be 88 eyes. To enhance precision the target sample was set at ≥100 participants. Ultimately, 122 healthy Saudi adults were recruited, exceeding the minimum requirement and providing greater statistical robustness. This sample size is comparable to those used in several previous studies, such as Al-Arfaj et al. (10; *n* = 96) and Yu et al. (14; *n* = 108), which strengthens the generalizability of our findings.

### 2.3 Participants

Eligible participants were Saudi aged 18 to 40 years with best-corrected visual acuity (BCVA) of 0.0 logMAR or better and no history of ocular disease. Exclusion criteria included systemic diseases with known ocular manifestations (e.g., diabetes mellitus), prior ocular surgery or trauma, contact lens wear within the preceding 3 weeks, current use of ocular medications, or corneal/media opacities affecting image acquisition or measurements. To avoid statistical bias arising from inter-eye correlation, only one eye per participant was selected randomly using an alternating sequence and included in the study, as recommended by Armstrong (18). The time of examination was between the 8:00 a.m. to 2:00 p.m.

### 2.4 Ophthalmic examination procedures

#### 2.4.1 Visual acuity and contrast sensitivity

Monocular distance visual acuity was measured using a standardized Early Treatment Diabetic Retinopathy Study (ETDRS) chart (Precision Vision, La Salle, IL, USA) at 4 meters under photopic conditions, with results recorded in logarithm of the minimum angle of resolution (logMAR) notation. Contrast sensitivity was assessed using the Mars Letter Contrast Sensitivity Test (Mars Perceptrix Corporation, Chappaqua, NY, USA) under controlled room illumination, and scores were calculated according to the manufacturer’s protocol.

#### 2.4.2 Refractive error measurement

Objective non-cycloplegic refraction was obtained using an autorefractor (ARK-1, NIDEK Co., Ltd., Gamagori, Japan). Refractive status was expressed not only as spherical equivalent refraction (SER) but also in vector components following the power vector method. Specifically, the spherical equivalent was represented as M, while astigmatic errors were decomposed into the Jackson cross-cylinder components: J0 (orthogonal/with-the-rule vs. against-the-rule astigmatism) and J45 (oblique astigmatism). These standardized vector parameters allow for a more comprehensive assessment of refractive error as suggested by Thibos et al. (19) in relation to corneal biomechanical and endothelial indices.

#### 2.4.3 Corneal biomechanical assessment

Corneal biomechanical parameters were measured with the Ocular Response Analyzer (ORA; Reichert Technologies, Depew, NY, USA) to quantify corneal hysteresis (CH), corneal resistance factor (CRF), Goldmann-correlated intraocular pressure (IOPg), and corneal-compensated intraocular pressure (IOPcc). For each eye, three valid readings were obtained by a single experienced examiner, and the mean value was used for analysis. All ORA measurements met the device’s internal waveform quality acceptance criteria. Only measurements with acceptable waveform quality were included. While no standardized cutoff exists, prior studies indicate that higher waveform scores are associated with improved reliability, with values ≥7 commonly considered optimal (20).

#### 2.4.4 Corneal endothelial morphology

Central corneal thickness and endothelial morphology was evaluated using a non-contact specular microscope (CEM-530, NIDEK Co., Ltd., Gamagori, Japan). Parameters extracted included endothelial cell density (ECD, cells/mm^2^), average cell size (AVG, μm^2^), minimum and maximum cell size (MIN, MAX, μm^2^), cell count (NUM), standard deviation of cell area (SD), coefficient of variation in cell size (CV, %), and percentage of hexagonal cells (HEX, %) and Central corneal thickness (CCT, μm) measured by the device’s optical pachymetry module. Central corneal images were captured three times per eye under standardized lighting conditions, and automated endothelial analysis was performed using the instrument’s built-in analysis software. Automated cell recognition was reviewed and manually corrected when necessary. For each parameter, the mean of the three acceptable measurements was used for analysis.

#### 2.4.5 Comparative analysis

For contextual benchmarking, mean CH and CRF values were compared with previously published international cohorts selected based on methodological similarity (ORA-based measurements in healthy populations). One-sample t-tests were used to compare the present cohort against reported means. These comparisons were exploratory and not part of a formal systematic analysis.

#### 2.4.6 Statistical analysis

All statistical analyses were performed using SPSS Statistics version 16.0 (IBM Corp., Armonk, NY, USA). Data normality was assessed using the Shapiro–Wilk test. All variables demonstrated normal distributions (*p* > 0.05), permitting parametric analyses. Descriptive statistics are reported as mean ± standard deviation.

Gender differences were evaluated using independent-samples t-tests. Refractive group comparisons (myopia, emmetropia, hyperopia) were performed using one-way analysis of variance (ANOVA) with Tukey post-hoc correction for multiple comparisons. Associations between corneal biomechanical parameters and endothelial or refractive variables were assessed using Pearson correlation coefficients. Correlation strength was interpreted as weak (|*r*| < 0.30), moderate (0.30 ≤ |*r*| < 0.50), and strong (|*r*| ≥ 0.50).

To identify independent predictors of ORA parameters (CH, CRF, IOPg, IOPcc), stepwise multiple linear regression was performed. Central corneal thickness, endothelial morphology metrics, and refractive components were entered simultaneously, and multicollinearity was assessed using variance inflation factors (VIF). Standardized beta coefficients (*β*) were used to compare the relative magnitude of independent predictors within each model. Because regression effect sizes are context-dependent and influenced by model composition, no rigid categorical thresholds were applied; instead, predictors were interpreted comparatively based on the magnitude of standardized β values and statistical significance. Statistical significance was defined as *p* < 0.05.

## 3 Results

A total of 122 participants (100 females and 22 males, 62 were right eyes and 60 were left eyes) were recruited in this study. The study evaluated a range of visual function parameters, including VA, CS, and refractive components expressed as power vector components (M, J0, and J45). Across the sample, mean values for these outcomes were closely aligned between genders, with minimal differences observed in age (*p* < 0.05). Overall, the findings suggest a largely homogeneous visual performance profile among the participants, regardless of gender. All other measured parameters, including ORA outcomes, and specular microscopy metrics, showed no statistically significant differences between genders (*p* > 0.05). Although, the male participants were observed to have lower scores in CH, CRF, ECD, and MAX but did not reach the statistically significant level. The descriptive statistics and gender comparisons are shown in Table 1.

**Table 1:** Mean ± SD and independent *t*-test results for all measured variables by gender.

Variable	Combined (mean ± SD)	Female (mean ± SD)	Male (mean ± SD)	*t*-statistic	*p*-value
Age (years)	22.11 ± 4.88	21.61 ± 4.26	24.36 ± 6.74	−2.45	0.016^a^
M (D)	−0.78 ± 1.77	−2.30 ± 2.37	−2.16 ± 2.41	−0.21	0.83
J0 (D)	0.25 ± 0.34	−0.06 ± 0.33	−0.04 ± 0.35	−0.19	0.85
J45 (D)	0.01 ± 0.19	0.02 ± 0.25	0.01 ± 0.28	0.11	0.92
CS (Log)	1.61 ± 0.04	1.61 ± 0.05	1.61 ± 0.01	0.58	0.56
ORA: CH (mmHg)	11.12 ± 2.09	11.21 ± 2.07	10.71 ± 2.18	1.00	0.31
ORA: CRF (mmHg)	11.23 ± 2.26	11.37 ± 2.17	10.61 ± 2.58	1.32	0.15
ORA: IOPcc (mmHg)	15.80 ± 3.67	15.84 ± 3.83	15.60 ± 2.87	0.27	0.78
ORA: IOPg (mmHg)	16.07 ± 3.76	16.25 ± 3.77	15.27 ± 3.70	1.11	0.27
Specular: AVG (μm^2^)	360.15 ± 31.23	357.62 ± 29.20	371.64 ± 37.84	−1.93	0.06
Specular: ECD (cells/mm^2^)	2796.62 ± 230.79	2814.60 ± 225.63	2714.91 ± 241.57	1.85	0.06
Specular: CCT (μm)	561.70 ± 34.13	563.25 ± 32.19	554.68 ± 42.03	1.07	0.29
Specular: CV (%)	27.38 ± 4.49	27.53 ± 4.56	26.68 ± 4.19	0.80	0.42
Specular: HEX (%)	67.67 ± 5.01	67.39 ± 5.22	68.95 ± 3.72	−1.33	0.19
Specular: MAX (μm^2^)	1100.13 ± 261.68	1103.60 ± 273.37	1084.36 ± 204.74	0.31	0.76
Specular: MIN (μm^2^)	140.88 ± 13.93	140.42 ± 13.76	142.95 ± 14.82	−0.771	0.44
Specular: NUM (count)	136.65 ± 40.98	133.73 ± 42.31	149.91 ± 31.87	−1.69	0.09
Specular: SD (μm^2^)	92.50 ± 17.27	92.22 ± 17.26	93.77 ± 17.69	−0.38	0.70

D, diopters; MARS, Mars Letter Contrast Sensitivity Test; ORA, Ocular Response Analyzer; CH, corneal hysteresis; CRF, corneal resistance factor; IOPcc, corneal-compensated intraocular pressure; IOPg, Goldmann-correlated intraocular pressure; ECD, endothelial cell density; CCT, corneal thickness; CV, coefficient of variation; HEX, hexagonality percentage; MAX, maximum cell size; MIN, minimum cell size; NUM, total number of cells; SD, standard deviation. ^a^Statistically significant (*p* < 0.05).

### 3.1 Differences across refractive groups

When assessing the Person’s relationship between ORA metrics and refractive/visual parameters, no significant associations were observed. No statistically significant associations were identified between corneal biomechanical parameters (CH, CRF, IOPg, IOPcc) and refractive or visual metrics (M, J0, J45, CS), with all correlation coefficients were weak (|r| ≤ 0.074, *p* > 0.41). Similarly, all endothelial morphology indices from specular microscopy showed negligible correlations with the same visual parameters (|r| ≤ 0.042, *p* > 0.66). These findings suggest that, within this cohort, corneal biomechanics and endothelial morphology were independent of refractive status and CS.

Analysis of variance (ANOVA) revealed a significant difference in Goldmann-correlated intraocular pressure (IOPg) between refractive categories (*F* = 4.560, *p* = 0.013), with Tukey’s post-hoc analysis showing higher values in hyperopes compared to myopes (mean difference = 2.581, *p* = 0.014). No statistically significant differences were found for CH, CRF, or corneal-compensated IOP (IOPcc) across refractive groups. Similarly, all specular microscopy parameters showed no significant intergroup differences (all *p* > 0.05).

### 3.2 Correlations between ORA metrics and specular microscopy parameters

**Figure 1:**
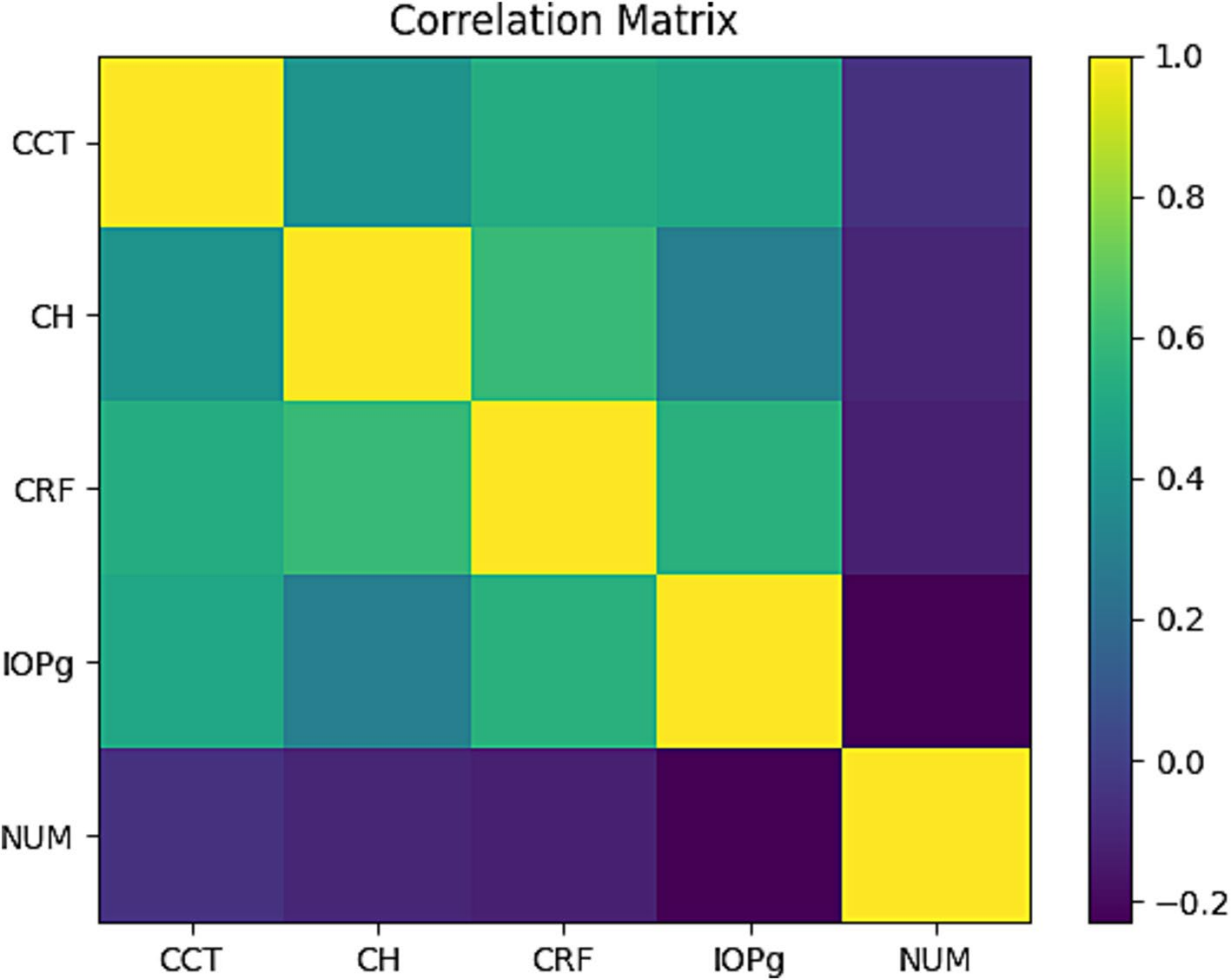
Correlation heatmap showing Pearson correlation coefficients between corneal biomechanical parameters and corneal endothelial metrics. Color intensity reflects correlation magnitude, with warmer colors indicating positive correlations and cooler colors indicating negative correlations. CH, corneal hysteresis; CRF, corneal resistance factor; IOPg, Goldmann-correlated intraocular pressure; IOPcc, corneal-compensated intraocular pressure; ECD, endothelial cell density (cells/mm^2^); CCT, central corneal thickness (μm); NUM, endothelial cell number (cell count); CV, coefficient of variation in endothelial cell size (%); AVG, average endothelial cell area (m^2^); SD, standard deviation of endothelial cell area (m^2^); HEX, percentage of hexagonal cells (%); MAX, maximum endothelial cell area (m^2^); MIN, minimum endothelial cell area (m^2^).

The cross-correlation analysis between the ORA metrics and the corneal specular microscopy parameters revealed several significant associations (Figure 1). In details, when ORA–specular metrics relationships were investigated, the highest observed association was between CRF and CCT showing a moderate positive correlation (*r* = 0.53, *p* < 0.0001), followed by IOPg (*r* = 0.50, *p* < 0.0001). The CH demonstrated a moderate positive correlation with CCT (*r* = 0.40, *p* < 0.0001). The IOPcc and IOPg showed a small but statistically significant negative correlation with NUM (*r* = −0.21, *p* = 0.021, *r* = −0.23, *p* = 0.011, respectively). Other ORA–specular relationships, were weak and statistically non-significant.

**Figure 2:**
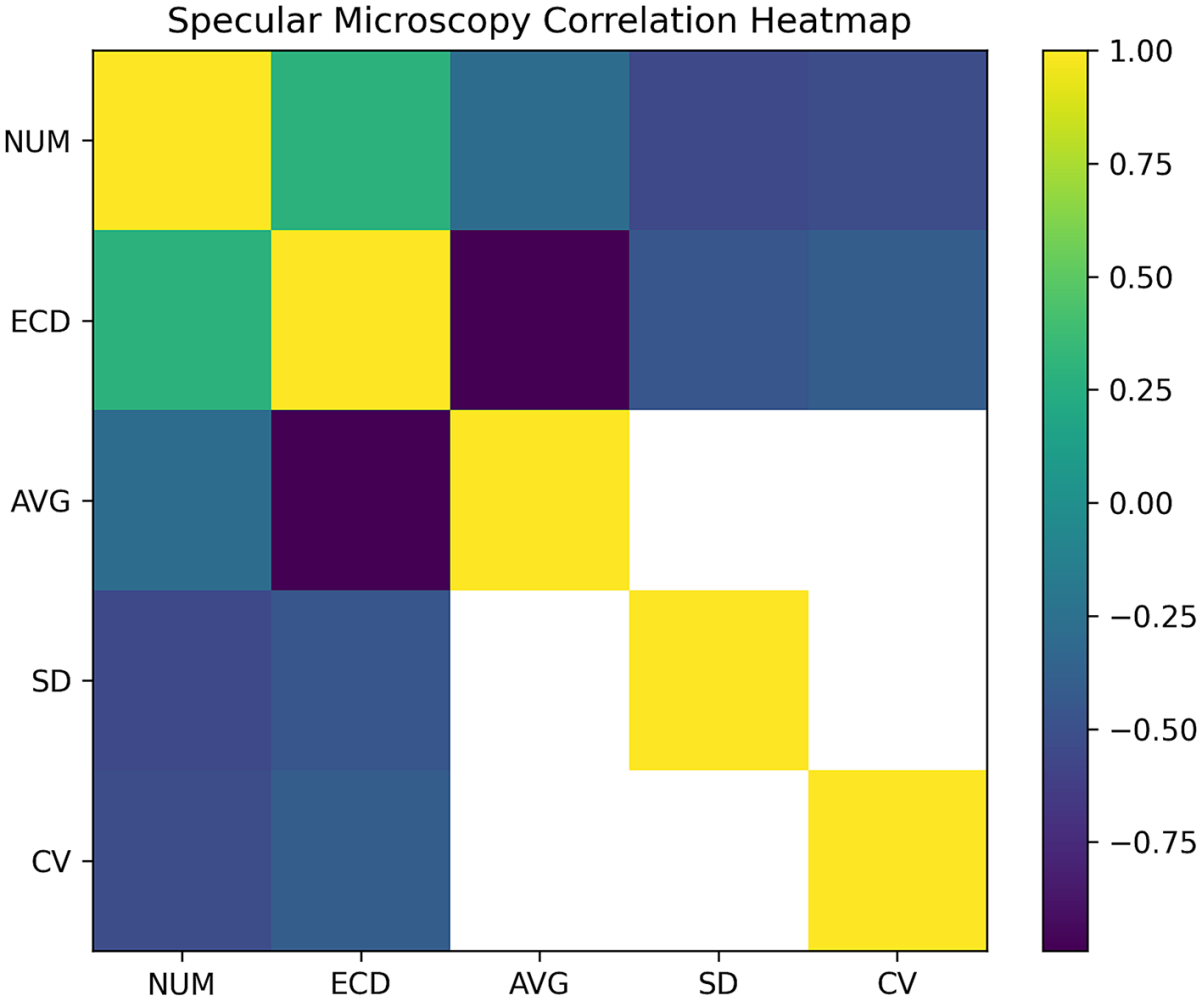
Correlation heatmap of specular microscopy parameters in this study cohort. Color intensity represents the magnitude and direction of correlations, with warmer colors indicating positive associations and cooler colors indicating negative associations. White cells indicate non-significant correlations (*p* > 0.05). Abbreviations: CH, corneal hysteresis; CRF, corneal resistance factor; IOPg, Goldmann-correlated intraocular pressure; IOPcc, corneal-compensated intraocular pressure; ECD, endothelial cell density (cells/mm^2^); CCT, central corneal thickness (m); NUM, endothelial cell number (cell count); CV, coefficient of variation in endothelial cell size (%); AVG, average endothelial cell area (μm^2^); SD, standard deviation of endothelial cell area (μm^2^); HEX, percentage of hexagonal cells (%); MAX, maximum endothelial cell area (μm^2^); MIN, minimum endothelial cell area (μm^2^).

The correlation analysis within the specular microscopy parameters revealed several notable associations (Figure 2). Specifically, A mild but significant positive correlation was observed between NUM and ECD (*r* = 0.285, *p* = 0.0015), while NUM was inversely correlated with both the AVG (*r* = −0.29, *p* = 0.0028) and the indices of variability, including the SD (*r* = −0.55, *p* < 0.0001) and the CV (*r* = −0.52, *p* < 0.0001). These findings indicate that higher cell counts are associated with more uniform cell morphology. In contrast, ECD demonstrated an almost perfect negative correlation with AVG (*r* = −0.990, *p* < 0.0001), reflecting the expected inverse mathematical relationship between these two parameters. Moreover, ECD was positively correlated with NUM (*r* = 0.285, p = 0.0015) and inversely related to SD (*r* = −0.46, *p* < 0.0001) and CV (*r* = −0.40, *p* < 0.0001), reinforcing the consistency of these relationships.

### 3.3 Predictors of ORA parameters

Across models, the CCT consistently emerged as the strongest predictor, showing significant positive associations with CRF (*β* = 0.377, *p* = 0.02) and IOPg (*β* = 0.370, *p* = 0.01), and near-significant trends for CH and IOPcc. Stepwise regression confirmed CCT’s influence, with additional contributions from endothelial metrics and refractive status.

For CH, only CCT remained significant (*β* = 0.024, *p* < 0.001), indicating that thicker corneas were associated with higher CH values. For CRF, both CCT (*β* = 0.036, *p* < 0.001) and endothelial cell density (*β* = −0.002, *p* = 0.04) were retained. The IOPg was predicted by CCT (*β* = 0.045, *p* < 0.001), endothelial cell count (*β* = −0.032, *p* < 0.001), spherical equivalent M (*β* = −0.512, *p* = 0.003), and coefficient of variation (*β* = −0.218, *p* = 0.007). IOPcc was influenced by endothelial cell count (*β* = −0.039, *p* < 0.001), cell area variability (*β* = −0.078, *p* < 0.001), and M (*β* = −0.400, *p* = 0.028) (Table 2).

**Table 2:** Multivariate predictors of ORA-derived biomechanical parameters.

Predictor	CH	CRF	IOPg	IOPcc
CCT	*β* = 0.024, *p* < 0.001	*β* = 0.036, *p* < 0.001	*β* = 0.045, *p* < 0.001	–
ECD	–	*β* = −0.002, *p* = 0.040	–	–
NUM	–	–	*β* = −0.032, *p* < 0.001	*β* = −0.039, *p* < 0.001
M	–	–	*β* = −0.512, *p* = 0.003	*β* = −0.400, *p* = 0.028
CV	–	–	*β* = −0.218, *p* = 0.007	*β* = −0.078, *p* < 0.001

CH, corneal hysteresis; CRF, corneal resistance factor; IOPg, Goldmann-correlated intraocular pressure; IOPcc, corneal-compensated intraocular pressure; CCT, central corneal thickness; ECD, endothelial cell density; NUM, endothelial cell number; M, spherical equivalent refraction; CV, coefficient of variation of endothelial cell size. “–” indicates the variable was not retained in the final stepwise regression model.

Collectively, these findings highlight the dominant role of corneal thickness, with supplementary effects from endothelial morphology and refractive profile, in determining biomechanical and pressure-related ORA parameters. Endothelial cell density and spherical equivalent demonstrated weak, non-significant trends in the remaining models, while CS and astigmatic components (J0, J45) contributed minimally.

### 3.4 Ethnic variations in corneal biomechanical parameters

**Figure 3:**
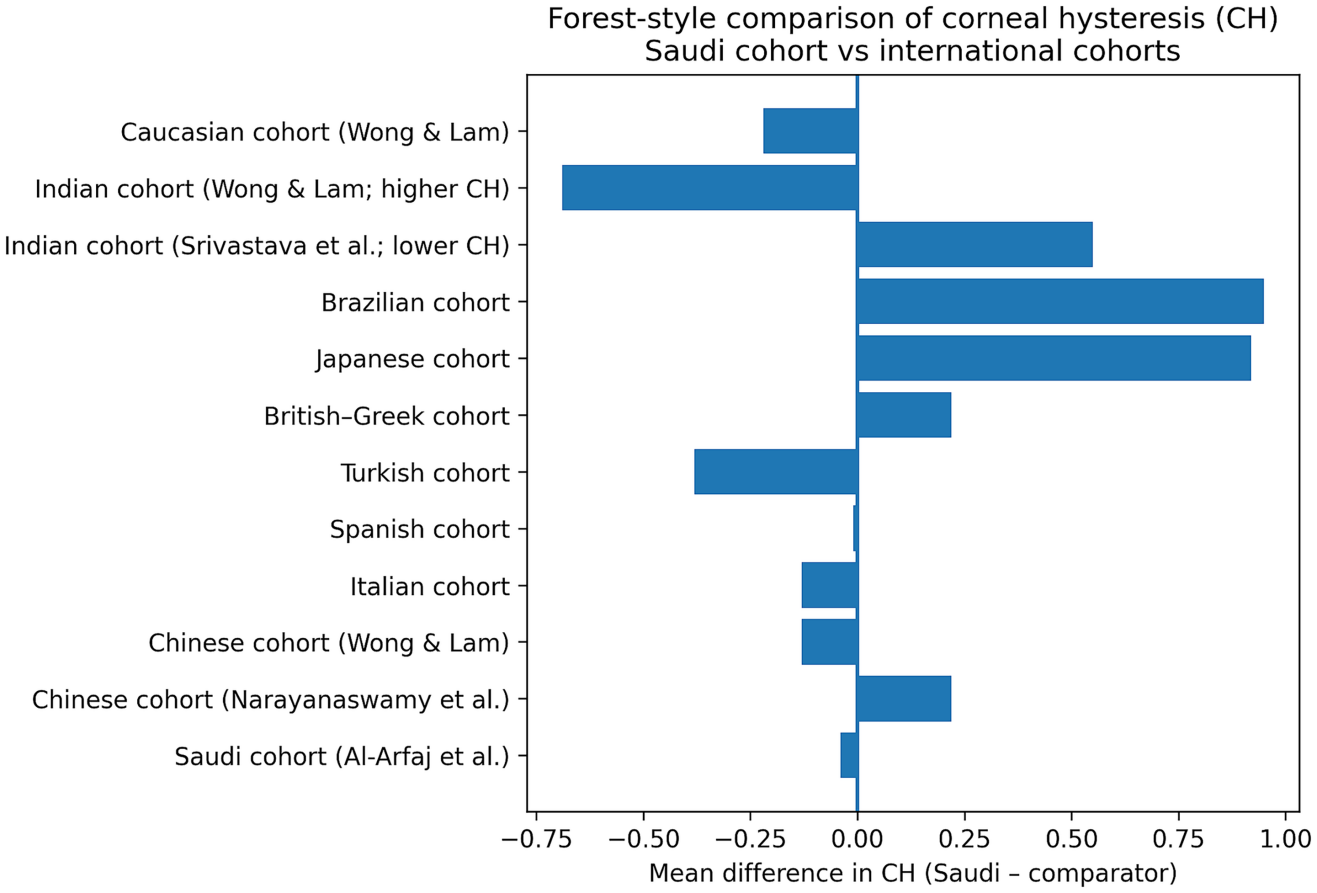
Forest-style plot of mean differences in corneal hysteresis (CH) between the Saudi cohort and international populations.

For contextual benchmarking, the mean corneal hysteresis (CH) and corneal resistance factor (CRF) values observed in the present Saudi cohort were compared with previously reported values from international populations. The CH and CRF values from the present cohort were compared to previously reported data across a wide range of populations using one-sample *t*-tests. For CH, this study outcomes (11.12 ± 2.09 mmHg) were not significantly different from those reported in a prior Saudi cohort (11.16 ± 2.11 mmHg; *t* = −0.21, *p* = 0.837) (10), Chinese populations (10.90 ± 1.40 mmHg; *t* = 1.17, *p* = 0.245 (9); and 11.25 ± 1.37 mmHg; *t* = −0.68, *p* = 0.497 (9)), Italian (11.25 ± 1.37 mmHg; *t* = −0.68, *p* = 0.497) (21), Spanish (11.13 ± 0.98 mmHg; *t* = −0.10, *p* = 0.919) (22), Turkish (11.50 ± 1.70 mmHg; *t* = −1.29, *p* = 0.198) (5), or British–Greek cohorts (10.90 ± 1.50 mmHg; *t* = 0.17, *p* = 0.866) (4). In contrast, significantly higher CH values were observed in our sample compared to Japanese (10.20 ± 1.30 mmHg; *t* = 4.87, *p* < 0.001) (11), Brazilian (10.17 ± 1.82 mmHg; *t* = 5.02, *p* < 0.001) (23), and one Indian cohort (10.57 ± 1.12 mmHg; *t* = 2.40, *p* = 0.018) (24), whereas our CH values were significantly lower than those reported in another Indian cohort (11.81 ± 1.33 mmHg; *t* = −4.57, *p* < 0.001) (16) and Caucasian individuals (11.34 ± 1.76 mmHg; *t* = −2.08, *p* = 0.038) (16) (Figure 3).

**Figure 4:**
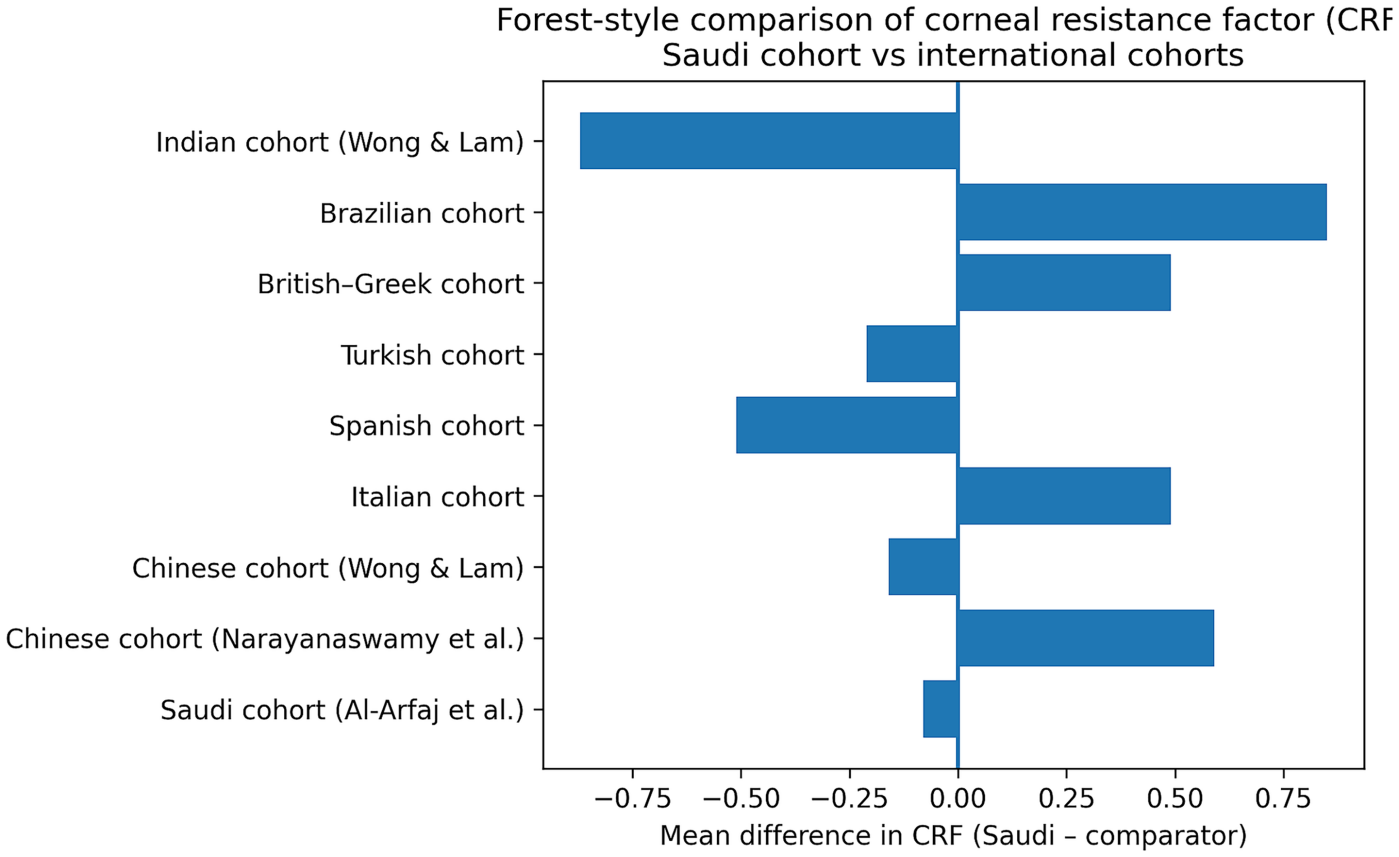
Forest-style plot of mean differences in corneal resistance factor (CRF) between the Saudi cohort and international populations. The data presented in this study are available on reasonable request from the corresponding author.

For CRF, our study (10.99 ± 2.41 mmHg) showed no significant differences from the Saudi cohort (11.07 ± 2.31 mmHg; *t* = −0.41, *p* = 0.682) (10), Chinese (10.40 ± 1.50 mmHg; *t* = 3.14, *p* = 0.002 (9); and 11.15 ± 1.48 mmHg; *t* = −0.52, *p* = 0.605 (16)), Italian (10.50 ± 1.70 mmHg; *t* = 1.77, *p* = 0.078) (21), Spanish (11.50 ± 1.10 mmHg; *t* = −1.87, *p* = 0.064) (22), Turkish (11.20 ± 1.40 mmHg; *t* = −0.60, *p* = 0.551) (5), and British–Greek cohorts (10.50 ± 1.70 mmHg; *t* = 1.77, *p* = 0.078) (4). However, significantly higher CRF values were observed in our sample compared to Brazilian (10.14 ± 1.80 mmHg; *t* = 3.56, *p* < 0.001) (23), while our CRF values were significantly lower than those reported in another Indian cohort (11.86 ± 1.87 mmHg; *t* = −3.31, *p* = 0.001) (16) (Figure 4).

## 4 Discussion

This study provides one of the first integrated evaluations of corneal biomechanics and endothelial morphology in a healthy Saudi population. Our findings demonstrate that central corneal thickness is the dominant predictor of ORA-derived parameters, while endothelial morphology contributes only modestly to pressure-related outcomes.

The Specular microscopy in the present study demonstrated that both endothelial cell density (ECD) and hexagonality (HEX) values were within the normal adult range, consistent with internationally reported normative data, including studies conducted in healthy adults from Thailand (ECD ≈ 2,907 cells/mm^2^, HEX ≈ 51.8%) and in Caucasian cohorts (ECD ≈ 2,752 cells/mm^2^, HEX ≈ 46%) (25, 26).

Importantly, our results demonstrated moderate positive correlations between CCT and CH, CRF, and IOPg, and regression analyses confirmed that CCT was the strongest independent predictor of all ORA-derived metrics. This finding is consistent with large population studies showing that corneal thickness explains more variance in biomechanical behaviour than age, refractive error, or IOP (3, 9).

By contrast, endothelial morphology exerted a more subtle influence: endothelial cell count (NUM) was independently associated with IOPg and IOPcc, suggesting that endothelial structural integrity modulates pressure-related biomechanical responses through its role in maintaining stromal hydration and viscoelastic damping. However, most endothelial parameters demonstrated weak or non-significant associations with CH and CRF, indicating that endothelial morphology plays a secondary rather than primary role in biomechanical regulation in healthy corneas.

Although the study’s sample was limited to younger adults (18–40 years), global evidence indicates that CH and CRF decline progressively with age due to biomechanical stiffening and endothelial attrition (4, 5, 27). In our cohort, male participants had lower mean values of CH, CRF, ECD, and MAX compared with females, though these differences did not reach statistical significance, consistent with mixed findings in the literature (21). Lifestyle and environmental factors may further modulate corneal biomechanics and endothelial health. Smoking has been shown to increase CH and CRF, likely through biochemical cross-linking of stromal collagen and altered tissue hydration (27, 28). Ultraviolet radiation, in contrast, accelerates endothelial cell loss and contributes to long-term corneal thinning and biomechanical instability (29). These influences are particularly relevant in Saudi Arabia, where high ambient UV exposure and outdoor occupations are common. Although these variables were not directly measured in the present study, they may contribute to inter-individual and population-level variation in corneal structure and function.

Our ANOVA results demonstrated that IOPg was significantly higher in hyperopes compared with myopes, while CH, CRF, and IOPcc did not differ significantly across refractive groups. This analysis was conducted across myopic, emmetropic, and hyperopic categories using one-way ANOVA with post-hoc testing. The absence of biomechanical differences contrasts with studies in highly myopic populations, where reduced CH and increased deformability are observed (12, 14). The relatively mild refractive errors in our cohort likely explain this discrepancy. Highlighting the need for stratification by refractive severity in future studies. Regression analyses further demonstrated that spherical equivalent (M) and astigmatic components (J0, J45) were weak, non-significant predictors of ORA parameters, and contrast sensitivity contributed minimally, reinforcing the consistency of CCT as the dominant factor.

Interestingly, the IOP was not retained as an independent predictor in the regression models, despite its established influence in previous studies. This discrepancy likely reflects the relatively narrow IOP range in our healthy cohort, which reduces statistical variability and limits its contribution in multivariate models. In contrast, CCT demonstrated greater variability and a stronger structural relationship with ORA parameters, thereby dominating the predictive models.

Our findings confirm that CH and CRF values in Saudi eyes are broadly comparable to those reported in prior Saudi cohorts (10), yet significant differences exist compared with international datasets. For instance, CH and CRF in our cohort were higher than in Japanese and Brazilian studies, but similar to Chinese values, supporting prior reports that ethnicity influences biomechanical indices (3, 4, 14). These inter-population differences likely reflect a combination of stromal collagen architecture, genetically determined variation in corneal thickness, and biomechanical remodelling driven by environmental exposures. Histological and tomographic studies demonstrate that collagen lamellar orientation, fibril diameter, and interweaving patterns differ across ethnic groups, leading to differences in corneal stiffness and viscoelastic damping even after CCT adjustment (3, 9). These structural variations have direct clinical relevance, as they influence corneal deformation under IOP and surgical stress. Clinically, our findings highlight the importance of accounting for central corneal thickness (CCT) when interpreting ORA-derived biomechanical parameters, as it demonstrated the strongest predictive influence. Endothelial morphology showed limited impact in healthy eyes but may become more relevant in pathological conditions such as Fuchs’ endothelial dystrophy or post-surgical corneas. Given the relatively high prevalence of keratoconus in Saudi Arabia (30, 31), and the established association between reduced corneal hysteresis (CH) and early ectatic disease (2, 32, 33), population-specific normative biomechanical data may improve screening accuracy and early detection in refractive surgery candidates.

This study has several limitations. The use of ORA-derived viscoelastic parameters without inclusion of dynamic elastic metrics limits the comprehensiveness of biomechanical assessment (3). In addition, the single-centre design, relatively young age range, and gender imbalance may affect generalizability. Refractive measurements were obtained under non-cycloplegic conditions; therefore, residual accommodative effects may have influenced refractive estimates. Environmental and lifestyle factors such as smoking and ultraviolet exposure were not directly assessed. Furthermore, waveform-score optimization strategies, as suggested by Yuhas et al. (34), were not implemented. Future multicentre studies incorporating broader age ranges, balanced demographics, and multimodal biomechanical assessment are warranted.

## 5 Conclusion

In summary, this study establishes foundational Saudi-specific data on the relationship between corneal biomechanics and endothelial morphology. Our findings highlight the dominant role of corneal thickness, confirm limited direct coupling between biomechanics and endothelial structure in healthy eyes, and underscore the importance of region-specific reference ranges. These insights carry direct implications for surgical planning, glaucoma assessment, and the refinement of corneal screening protocols in Saudi clinical practice.

## Data Availability

The raw data supporting the conclusions of this article will be made available by the authors, with reasonable request.

## Glossary

ARK-1: Autorefractor model used (NIDEK, Gamagori, Japan)
AVG: Average cell size (μm^2^)
BCVA: Best-corrected visual acuity
CCT: Central corneal thickness
CEM-530: Specular microscope model used (NIDEK, Gamagori, Japan)
CH: Corneal hysteresis
CRF: Corneal resistance factor
CS: Contrast sensitivity
CV: Coefficient of variation in cell size (%)
D: Diopters
ECD: Endothelial cell density (cells/mm^2^)
ETDRS: Early Treatment Diabetic Retinopathy Study
HEX: Percentage of hexagonal cells (%)
IOPcc: Corneal-compensated intraocular pressure
IOPg: Goldmann-correlated intraocular pressure
J0: Jackson cross-cylinder (with-the-rule vs. against-the-rule astigmatism)
J45: Jackson cross-cylinder (oblique astigmatism)
logMAR: Logarithm of the minimum angle of resolution
M: Spherical equivalent refraction
MARS: Mars Letter Contrast Sensitivity Test
MAX: Maximum cell size (μm^2^)
MIN: Minimum cell size (μm^2^)
NUM: Endothelial cell number (count)
ORA: Ocular Response Analyzer
SD: Standard deviation of cell area (μm^2^)
SPSS: Statistical Package for the Social Sciences
